# Changes in the Spectrum of Free Fatty Acids in Blood Serum of Dairy Cows during a Prolonged Summer Heat Wave

**DOI:** 10.3390/ani11123391

**Published:** 2021-11-27

**Authors:** Roman Mylostyvyi, Veerasamy Sejian, Olena Izhboldina, Olena Kalinichenko, Lina Karlova, Olena Lesnovskay, Natalia Begma, Oleh Marenkov, Vadym Lykhach, Svitlana Midyk, Nikolay Cherniy, Bogdan Gutyj, Gundula Hoffmann

**Affiliations:** 1 Department of Animal Products Processing Technology, Dnipro State Agrarian and Economic University, S. Efremov Str. 25, 49600 Dnipro, Ukraine; mylostyvyi.r.v@dsau.dp.ua (R.M.); kalynychenko.o.o@dsau.dp.ua (O.K.); 2 National Institute of Animal Nutrition and Physiology, Indian Council of Agricultural Research, Adugodi, Bangalore 560030, India; drsejian@gmail.com; 3 Department of Livestock Production Technology, Dnipro State Agrarian and Economic University, S. Efremov Str. 25, 49600 Dnipro, Ukraine; izhboldina.o.o@dsau.dp.ua (O.I.); lesnovska.o.v@dsau.dp.ua (O.L.); 4 Department of Animal Feeding and Breeding Technology, Dnipro State Agrarian and Economic University, S. Efremov Str. 25, 49600 Dnipro, Ukraine; karlova.l.v@dsau.dp.ua (L.K.); behma.n.a@dsau.dp.ua (N.B.); 5 Department of General Biology and Water Bioresources, Oles Honchar Dnipro National University, Gagarin av., 72, 49010 Dnipro, Ukraine; gidrobions@gmail.com; 6 Department of Technologies in Poultry, Pig and Sheep Breeding, Faculty of Livestock Raising and Water Bioresources, National University of Life and Environmental Sciences of Ukraine, Heroiv Oborony St. 12-b, Building 7-a, of. 215, 03041 Kyiv, Ukraine; vylykhach80@nubip.edu.ua; 7 Ukrainian Laboratory of Quality and Safety of Agricultural Products, National University of Life and Environmental Sciences of Ukraine, Heroiv Oborony Street, 15, 03041 Kyiv, Ukraine; svit.mid@gmail.com; 8 Department of Animal Hygiene and Veterinary Sanitation, State Biotechnological University, Alchevskykh Street, 44, 61002 Kharkiv, Ukraine; nycvas@ukr.net; 9 Department of Hygiene, Sanitation and General Veterinary Prevention, Faculty of public development and health, Stepan Gzhytskyi National University of Veterinary Medicine and Biotechnologies Lviv, Pekarska Str., 50, 79010 Lviv, Ukraine; bvh@ukr.net; 10 Department of Engineering for Livestock Management, Leibniz Institute for Agricultural Engineering and Bioeconomy, Max-Eyth-Allee 100, 14469 Potsdam, Germany

**Keywords:** dairy cow, blood serum, free fatty acids, heat stress

## Abstract

**Simple Summary:**

Heat stress leads to poor welfare, decreased productivity, and poor product quality. It is known that the content of fatty acids in the blood can reflect the physiological state of the body under normal and pathological conditions. They can be biomarkers for the state of biomembranes associated with inflammation and indicate the state of energy imbalance during chronic heat stress. They perform various functions in the body; therefore, the determination of the spectrum of free fatty acids can be used as biomarkers of these processes. The changes in the spectrum of free fatty acids in the blood serum of dairy cows revealed in our study will make it possible to better understand the physiological state of the organism and possibly indicate ways to maintain the health and milk productivity of animals under conditions of prolonged hyperthermia.

**Abstract:**

This experiment was conducted to study the effect of a prolonged hot period on the fatty acid (FA) composition in blood serum of dairy cows. Eighteen multiparous Holstein cows were randomly assigned to the hyperthermia group (HYP, *n* = 8) in August (summer season) and the control group (CON, *n* = 10) in October (autumn season). Blood from animals of the HYP group was collected in one heat wave, which was preceded by a long period of heat stress (HS, temperature-humidity index (THI ≥ 72)). Blood from cows of the CON group was collected under thermal comfort conditions (THI < 68). The spectrum of free fatty acids (FFA) in the blood serum was analyzed by gas chromatography. The concentration of FFA increased, including saturated FAs and monounsaturated FAs, in the blood serum of cows under conditions of prolonged HS. This was associated with the mobilization of FA into the bloodstream from adipose tissue, as a consequence of negative energy balance. An increase in the ratio of n-6/n-3 polyunsaturated FAs may indicate biomembrane dysfunction and adversely affect dairy cows. This study showed that prolonged periods of heat can affect the FA composition of blood. How much this leads to changes in the FA composition of milk and the quality of food products remains to be seen in further research.

## 1. Introduction

The role of fatty acids (FA) in a living organism is extremely diverse. This is closely related to the structure of these biologically active molecules, which have different carbon chain lengths and degrees of saturation. In particular, saturated fatty acids (SFA) are a source of energy and play an important role in the prevention of lipid oxidation of cell membranes. Polyunsaturated fatty acids (PUFA), which are part of biomembrane phospholipids, provide not only their structure but also regulate intracellular metabolism and create optimal conditions for the activity of multienzyme systems [1,2]. Separate FAs are precursors of many biologically active substances (prostaglandins, leukotrienes, thromboxanes, and many others) involved in the regulation of metabolic processes in humans and animals.

The concentration and composition of FAs vary significantly under various physiological and pathological conditions. The determination of their content in various biological substrates, including blood plasma, can be an important diagnostic tool [3]. This allows early detection of systemic disorders and diseases associated with lipid imbalance and hence improve the quality of treatment. Many researchers emphasize the importance of studying certain types of FAs as biological markers of metabolic homeostasis disorders and early detection of pathological conditions in the body [4,5].

The FA composition in the blood of cows is associated with the peculiarities of the functioning of the ruminant organism. It largely depends on the intensity of hydrogenation of unsaturated fatty acids in the rumen by microorganisms [6]. It also depends on oxidation, desaturation, and lengthening of fatty acids in body tissues [7] and their ratio in blood and adipose tissue (AT). Differences in blood lipid composition depend on breed characteristics, productivity, season, period, pregnancy, and lactation [8,9]. Some of the PUFAs are essential, such as the n-6 family on the basis of linoleic acid and the n-3 family on the basis of linolenic acid, since dairy cattle, like humans, cannot produce these FAs. They are an integral part of the diet, derived from plant sources or from animals that have received these PUFAs from plant sources. Despite the fact that inflammation can increase with the intake of some n-6 PUFAs and their further incorporation into membrane phospholipids, it is also possible to relieve inflammation by taking n-3 PUFAs [6]. The introduction of these PUFAs into the diet of livestock in order to influence the pathogenesis of diseases has attracted more and more attention of researchers in recent years [10].

Heat stress (HS) in dairy cows is associated with impaired homeostasis, metabolic disturbances, and the development of oxidative stress [11]. This results in reduced milk yield and changes of milk components, including lipids [5]. Some blood FAs are precursors of milk FAs or participate in de-novo lipid formation in secretory cells of the mammary gland. Therefore, the study of the spectrum of serum free FAs will be useful not only for the assessment of animal health and welfare [12,13] but also for the possible impact of HS on the quality of milk and dairy products [14], which is becoming increasingly important in the context of global warming. We hypothesized that prolonged periods of heat can affect the FA composition of the blood. Therefore, we studied the spectrum of serum FAs in dairy cows as possible biomarkers of the state of biomembranes and negative energy balance during prolonged seasonal heat stress compared with the serum FA profile of cows under conditions of thermal comfort in the autumn period.

## 2. Materials and Methods

### 2.1. Animals

This experiment was performed in accordance with the requirements for humane treatment of animals and approved by the Commission on Bioethics of the Institute of Biotechnology and Animal Health. Eighteen lactating Holstein multiparous cows (2nd or 3rd lactation) were assigned to one of two groups. Some cows were assigned to the hyperthermia group (HYP, *n* = 8) in August (summer season), and another part was assigned to the control group (CON, *n* = 10) in October (autumn season). Each group was formed randomly and consisted of different cows with 117 to 152 days in milk (DIM). Differences in DIM (LSM ± SE) between the HYP and CON groups (130.2 ± 3.13 and 130.5 ± 2.81, respectively) were not significant (*p* = 0.95). The average daily milk yield of cows (LSM ± SE) in the CON and HYP groups was 24.6 ± 0.45 kg and 24.8 ± 0.48 kg, respectively. The difference between the groups in milk yield was not significant (*p* = 0.96). The difference in percentage of milk fat between the CON group (3.79 ± 0.032) and the HYP group (3.49 ± 0.034) was significant (*p* ≥ 0.001). The percentage of milk protein in the CON group (3.30 ± 0.025) and HYP group (3.19 ± 0.027) also showed a significant difference (*p* = 0.037).

### 2.2. Keeping and Feeding Animals

The research was carried out on a commercial dairy farm in Ukraine located within the Dnipropetrovsk Oblast (48°28′44′′ N 35°36′46′′ E). In brief, dairy cows were kept loose in a naturally ventilated barn. Sand was used as bedding in the cubicles. Holstein cows were fed a corn silage-based total mixed ration (TMR) all year round. The diets were balanced in essential nutrients according to National Research Council guidelines [15]. The barn had a feeding alley and six water troughs, which were freely accessible. The housing conditions of the cows were described more detailed in our previously published study [16].

### 2.3. Weather Conditions

Heatwaves have been identified as a sequence of at least 5 consecutive hot days [17]. A hot day was defined as a day with a daily maximum air temperature (Tmax) exceeding the 95th percentile of all values within the analyzed period (from 13 June to 15 October 2018). In our case, the 95th percentile for Tmax was 30 °C. This was consistent with data from Tomczyk et al. [17], who indicated the 95th percentile for Tmax between 25 °C and 30 °C in their study for the eastern region of Europe (R3). Weather data from the nearest meteorological station were taken on the website of the Ukrainian Hydrometeorological Center, as it has been described previously [18]. Furthermore, the climate conditions in the barn were recorded with a thermohygrometer (Benetech GM 1360, Shenzhen Jumaoyuan Science and Technology Co., Ltd., Shenzhen, China).

**Figure 1:**
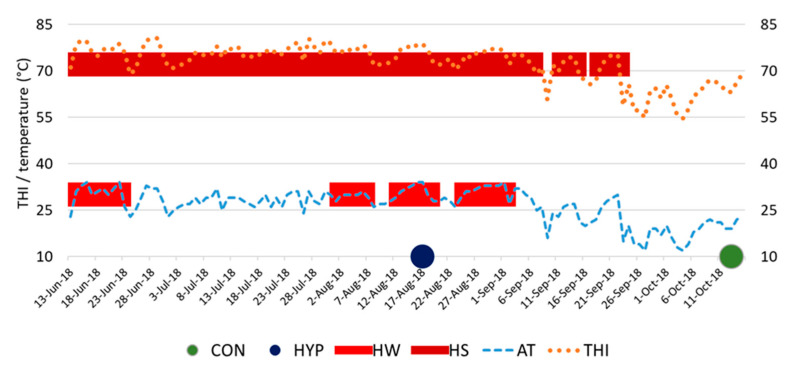
Environmental parameters during the experimental period. CON, control group of animals; HYP, group of animals in conditions of hyperthermia; HW, heat wave; HS, heat stress; AT, air temperature; THI, temperature-humidity index.

The temperature-humidity index (THI), calculated according to Kibler [19], served as an indicator of heat stress (HS) in cows. The THI limit at which most dairy cows could sense HS was 72 [20]. The weather conditions of the present study are illustrated in Figure 1.

### 2.4. Collection of Blood Samples and Analysis of Serum

Blood samples from both groups of cows were taken during the warmest time from 13:00 to 14:00 pm, with fixing the animals in headlocks near the feeding alley, after their return from milking. Blood samples (10 mL) were taken from the jugular vein of each cow into a clean dry tube without anticoagulants and were cooled afterwards. They were centrifuged at 3000× *g* for 20 min. Serum was separated into 1.5 mL clean, dried Eppendorf tubes (Eppendorf AG, Hamburg, Germany) and frozen at −20 °C until analysis.

The frozen serum was thawed overnight before testing. Free fatty acids in blood serum was determined by a chromatographic study using a hardware-software complex for medical research based on the Chromatec-Crystal 5000 gas chromatograph (Chromatec, Yoshkar-Ola, Russian Federation).

### 2.5. Equipment and Instrument Operation Mode during Research

The gas chromatograph was equipped with a flame ionization detector. The quartz capillary column (Restek, Bellefonte, PA, USA) was 60 m long, its inner diameter was 0.25 mm, the stationary phase type was FFAP, and film thickness was 0.25 μm.

The isothermal temperature of the thermostat was 140 °C, and the temperature of the evaporator and detector was 230 °C. The carrier gas was nitrogen, with a pressure of 1.8 atm at the column inlet. Carrier gas consumption was 2 mL/min, hydrogen 25 mL/min, and air 300 mL/min. The ratio of carrier gas flows to discharge and to column was 50:1.

### 2.6. KOH-Methylation of Lipids in Biological Substrates Containing Water (Blood Serum)

To 0.14 mL of the sample, 2 mL of hexane (30 °C) and 0.28 mL of KOH (Potassium hydroxide, 1 M in 70% methanol, 30 °C) were added, and the tubes were stirred for 2.5 min and kept in a water bath at 30 °C for 20 min, stirring occasionally. The reaction was stopped with 0.03 mL of acetic acid. Then, the methylated FAs that were in the hexane layer were analyzed on a gas chromatograph.

Quantitative identification of free FAs fractions, column calibration, and chromatogram calculation were performed using the method of normalization of peak areas and their fractions (described by Ichihara et al. [21]) according to the standards of FAs of Restek (Bellefonte, PA, USA). Samples were run in a single copy using negative controls.

### 2.7. Statistical Analysis

Statistical software package STATISTICA 10 (StatSoft, Inc., Tulsa, OK, USA) was used for statistical data processing. The distribution of almost all variational series did not meet the criteria of normality; therefore, the methods of nonparametric statistics were used in the further analysis. Data are presented as medians and interquartile ranges. Differences between the samples were determined by the Mann–Whitney U test and were considered significant at *p* < 0.05.

## 3. Results

### 3.1. Barn Climate Conditions

The climatic conditions during the experimental study time are shown in Table 1. Blood sampling from HYP animals occurred on the fifth day of a recurring heatwave that lasted from 13 August to 18 August 2018. This period was preceded by several more heat waves, the nearest lasting five days (from 2 August to 6 August) and a more distant heat wave lasting nine days (from 14 June to 22 June). The continuous period during which the dairy cows of the HYP group were in the HS state (THI ≥ 72) before the time of blood samplings was 45 days. At the time points of blood sampling of HYP cows, the minimum THI in the barn was 77.9 (Table 1).

In animals of the CON group, blood samples were taken on 13 October 2018 under thermal comfort conditions (THI = 63 at time points of blood sampling). It took 42 days from the last heat wave lasting eight days (from 25 August to 1 September) to the day of blood sampling from CON cows. Twenty-one days elapsed from the last day with heat stress conditions (THI ≥ 72) to the time of blood draw in the CON group.

### 3.2. The Spectrum of Serum Fatty Acids

Significant changes in the spectrum of FAs in blood serum are presented in Table 2. A significant increase in short-chain FAs was observed in animals of the HYP group. The concentration of caproic acid (C6:0) in serum was 19 times (*p* = 0.139) and butyric acid (C4:0) 83 times higher (*p* = 0.032) than in cows of the CON group.

It should be noted that SFAs with a carbon chain length of C8:0–C11:0 appeared in the blood serum of HYP animals, whereas they were not detectable in the serum of CON animals. The total content of it was 2.56 μg/μL (or 14.5% of the total content of free FAs of the HYP group).

However, despite a significant higher value in SFAs with short and medium carbon chain lengths, the overall level of SFAs in blood serum was less in the HYP group.

This was due to a lower concentration of myristic acid (3.5 times less, *p* = 0.01), pentadecanoic acid (1.5 times less, *p* > 0.05), and especially heptadecanoic acid (3.9 times less, *p* > 0.05) in HYP compared to CON. At the same time, the share of C17:0 acid in the total amount of free fatty acids in the blood serum of cows decreased from 55.7% (in CON) to 15.2% in HYP. The concentration of long-chain SFAs was also lower under hot conditions; arachidic acid was 2.3 times less (*p* > 0.05) and heneicosanoic acid 4.1 times less (*p* = 0.044) as in the CON group.

In animals of the HYP group, the lower concentration of monounsaturated FAs in the blood serum compared to CON (*p* < 0.05) was due to *cis*-9 C14:1 (2.0 times less), *cis*-9 C16:1 (4.3 times less), *cis*-10 C17: 1 (2.0 times less), as well as *trans*- and *cis*-isomers C18:1 (2.0 and 1.9 times less, respectively). In addition, no eicosanoic acid (*cis*-11 C20:1) was detected in the serum of the HYP group. However, due to long-chain PUFAs, the level of unsaturated FAs in the total spectrum of free FAs in HYP cows was 14.7% higher than in CON cows (Table 3).

In particular, the concentration of *trans*-9,12 and *cis*-9,12 isomers of linoleic acid (C18:2n-6) was 5.3 times higher in HYP than in CON animals. The content of α-linolenic acid (*cis*-9,12,15 C18:3n-3) was 1.5 times higher although the concentration of γ-linolenic acid (*cis*-6,9,12 C18:3n-6) in blood serum was 2.3 times lower in HYP compared to CON animals. In general, the proportion of polyunsaturated FAs decreased from 24.4% in the HYP to 11.6% in the CON group. Summarizing, we note that under conditions of hyperthermia, the ratio of UFA/SFA and the ratio n-6/n-3 was a pronounced tendency to increase in the blood serum of dairy cows, mainly due to an increase in the concentration of linoleic acid.

## 4. Discussion

Heat stress (HS) in dairy cows occurs when the ability to dissipate heat exceeds the range established for normal activity and the body makes adjustments to avoid physiological dysfunction [22]. Compensatory reactions of the animal organism to HS are manifested in behavioral changes (e.g., lying behavior, feed intake) as well as in clinical and metabolic reactions, which can affect their productivity. Reduced feed intake combined with increased costs of maintaining homeostasis during HS lead to a negative energy balance (NEB) in dairy cows. The release of nutrients from the tissues of the system is a key mechanism that cows with a NEB use to maintain lactation while reducing nutrient intake from the diet [11,23].

Lipids are usually deposited in adipose tissue (AT), but numerous studies have also shown that AT is involved in the regulation of metabolic processes through the control of the level of lipids and free fatty acids (FFA) in the blood plasma. More and more evidence are emerging to support the concept that peptides and hormones produced in AT are an important component of endocrine regulation of homeostasis of carbohydrates and lipids [4].

Determination of serum FAs can be used as potential biomarkers for monitoring animal stress and welfare [12]. In studies on lactating cows [13], it was concluded that FAs have high sensitivity and specificity in diagnosing HS status. The increased content of short-chain FAs (especially butyric acid) in the serum of HYP cows may be a consequence of their release from AT as a result of intense lipolysis [24]. This is due to the fact that fat decomposition compensating for energy shortage, which was an adaptive response to NEB [12]. This was also indicated by a significant increase in the serum of monounsaturated FAs since it is known that lipids of AT of ruminants are characterized by a high content of SFA and monounsaturated FAs [25].

In a recent review, Raphael and Sordillo [6] reported in-vitro experiments in which saturated fatty acids (SFAs) can directly activate pro-inflammatory signaling pathways and enhance the expression of pro-inflammatory cytokines, mimicking the action of endotoxins. When FA derived from AT are mobilized into the bloodstream, phospholipid biosynthesis must compete for the PUFA substrate with ketogenesis, β-oxidation, and secretion of milk lipids. Despite this competition, there is evidence that FA derived from AT alter the phospholipid composition in vascular endothelial tissue and also induce inflammation via lipid mediators [6]. Therefore, increased concentrations of individual short-chain FAs in our study should be considered as a negative effect on the organism of cows under conditions of hyperthermia.

The pronounced tendencies revealed by us in an increase in the PUFA content and the ratio of UFA/SFA may indicate a dysfunction of biomembranes during HS. The PUFA content of phospholipids influences inflammation through several mechanisms, including membrane fluidity, lipid raft formation, and receptor function [6]. Many facts indicate that HS in cows is accompanied by oxidative stress in the animal’s organisms. Oxidative stress, for example, occurs when there is an imbalance between the production of oxygen radicals during periods of high metabolic demand and the reduced capabilities of the host’s antioxidant defenses. During oxidative stress, reactive oxygen species (ROS) can modify PUFAs associated with cellular membranes, resulting in the biosynthesis of oxidized products called oxylipids [2]. Since several PUFAs, including linoleic acid, are the predominant substrates for oxylipid biosynthesis [6], a pronounced tendency to their increase may be the reason for a possible aggravation of oxidative stress of HYP cows.

It is known from the literature that HS negatively affects the health of the rumen due to a decrease in feed intake and hence less chewing and reduced buffering agents coming into the rumen. The less effective absorption of end products of digestion, including volatile fatty acids due to redistribution of blood flow to the periphery, leads to rumen acidosis, which indirectly increases the risk of negative side effects (for example, laminitis, decreased milk fat, etc.) [11]. Our study did not investigate the dry matter intake (DMI) of the diet intake during HS, and the TMR was also not chemically analyzed. However, a decrease in DMI can be expected since a result of the receipt of a saturation signal through the oxidation of FFA metabolites in the liver [11]. Despite the fact that animals of different groups in our study had a similar physiological status and received the corresponding TMR for mid-lactation cows, the feed factor could have brought its own limitations to our work, as seasonality can affect the chemical composition of TMR [26].

It is known that an increase in the concentration of FFA in blood serum during excessive lipolysis adversely affects metabolism and milk production [27]. This was confirmed by the significantly lower content of milk fat and protein in cows in the HYP group (see Section 2.1). The fact that cows exposed to HS are unable to harness this “shift” in energy metabolism to support milk production also may indicate direct HS not mediated by feed intake [11]. It should be noted that the differences in milk fat and protein content in our case may be associated with changes in the content of serum FAs, as individual FAs either enter the milk directly from the blood or take part in its formation de novo [28].

A high ratio of n-6/n-3 PUFA in plasma or a high omega-3 index is associated with the occurrence of metabolic diseases, inflammation, and other disorders [29]. In our case, an increase in the ratio of n-6/n-3 PUFAs in the blood serum of cows with pro-longed HS (compared with the CON group) may indicate changes in the structure and fluidity of biomembranes and may also indicate a violation of the implementation of membrane-dependent functions of cellular and subcellular structures [30].

It is noteworthy that the ratio of n-6 and n-3 PUFAs in tissue phospholipids is a direct reflection of the composition of the diet since livestock cannot synthesize n-3 PUFAs from n-6, because they lack specific desaturase enzymes [10]. It is also noteworthy that modern diets of intensively farmed ruminants, as in our case, may be partly responsible for the increased ratio of n-6 to n-3 PUFAs in phospholipids and the effects this has on the severity and duration of inflammation [6], even without exposure to high temperatures.

The n-6/n-3 PUFA ratio is considered an index of healthy balance in the body [30] to the same extent as the lower ratio of n-6/n-3 in milk causes a more favorable product composition [31]. It is quite possible to assume that a lower fat content in cow’s milk during prolonged HS may also be accompanied by an increase in the omega-3 index since all the FAs in the milk fat starting from C18:0 are derived from the arterial blood [28]. It is known that inflammation can be exacerbated by ingesting certain dietary FAs, such as some n-6 PUFAs, and then incorporating them into membrane phospholipids. The inflammation, however, can be relieved by taking other FAs, such as n-3 PUFAs [6]. Therefore, do not underestimate the possible impact of dietary FAs on health, which are earning more attention lately. The revealed trends in the content of serum FAs in cows under prolonged HS in this study require further confirmation. The limited number of serum samples and running them in simple rather than duplicate form could also be a limitation in our work.

## 5. Conclusions

Hot weather caused a change in the FA composition in the blood serum of dairy cows. An increase in the content of saturated FAs and monounsaturated FAs as a result of AT mobilization may indicate the development of NEB in animals. Biomembrane dysfunction and oxidative stress associated with increased UFA/SFA and n-3/n-6 PUFA ratios can affect the health of dairy cows during prolonged HS. Further research is warranted to confirm the identified trends in serum FAs.

## Figures and Tables

**Table 1:** Climate conditions in the barn under conditions of hyperthermia (HYP) and during the control period (CON), THI, temperature-humidity index.

Indicator	HYP			CON		
	Median	Min	Max	Median	Min	Max
	Microclimatic parameters during blood sampling ^1^					
Temperature, °C	34.0	33.0	34.0	19.0	19.0	19.0
Relative humidity, %	26.0	25.0	28.0	35.0	30.0	35.0
THI	78.4	77.9	78.6	63.1	62.9	63.1
	Microclimatic parameters on the day of blood sampling					
Temperature, °C	25.5	19.0	34.0	9.0	4.0	19.0
Relative humidity, %	46.0	25.0	83.0	58.5	30.0	81.0
THI	71.8	65.4	79.1	50.0	41.2	63.1
	Microclimatic parameters on the day before blood sampling					
Temperature, °C	25.5	21.0	34.0	10.5	8.0	19.0
Relative humidity, %	41.0	23.0	65.0	57.5	32.0	82.0
THI	71.6	66.4	78.0	52.2	47.6	63.2
	Microclimatic parameters during the week before blood sampling					
Temperature, °C	24.0	14.0	34.0	11.0	3.0	22.0
Relative humidity, %	47.0	23.0	83.0	63.0	25.0	93.0
THI	70.0	57.3	78.7	52.6	38.9	67.1

^1^ Blood samples were taken from cows from 13:00 to 14:00.

**Table 2:** Spectrum of free fatty acids (μg/μL) in blood serum of dairy cows under conditions of hyperthermia (HYP) or in the control group (CON), presented as median (25th and 75th percentile values in brackets).

Fatty Acid	Group of Animals	
	HYP (*n* = 8)	CON (*n* = 10)
Saturated fatty acids (SFA)		
Butyric acid (C4:0)	0.083 (0.054; 2.945) ^a^	0.001 (0.001; 0.001) ^b^
Caproic acid (C6:0)	0.939 (0.056; 1.163) ^a^	0.049 (0.049; 0.049) ^a^
Caprylic acid (C8:0)	0.897 (0.464; 1.124)	BDL
Capric acid (C10:0)	0.008 (0.005; 0.014)	BDL
Undecanoic acid (C11:0)	0.007 (0.001; 0.007)	BDL
Lauric acid (C12:0)	0.008 (0.004; 0.015) ^a^	0.006 (0.004; 0.027) ^a^
Tridecanoic acid (13:0)	0.002 (0.001; 0.003) ^a^	0.002 (0.001; 0.002) ^a^
Myristic acid (C14:0)	0.002 (0.001; 0.003) ^a^	0.007 (0.005; 0.010) ^b^
Pentadecanoic acid (C15:0)	0.010 (0.005; 0.182) ^a^	0.15 (0.058; 0.178) ^a^
Palmitic acid (C16:0)	0.013 (0.008; 0.019) ^a^	0.01 (0.007; 0.015) ^a^
Margarinic acid (C17:0)	0.203 (0.033; 0.555) ^a^	0.799 (0.195; 1.068) ^a^
Stearic acid (C18:0)	0.039 (0.027; 0.092) ^a^	0.036 (0.018; 0.078) ^a^
Arachinic acid (C20:0)	0.019 (0.016; 0.038) ^a^	0.043 (0.032; 0.061) ^a^
Geneicosanoic acid (C21:0)	0.054 (0.032; 0.070) ^a^	0.222 (0.101; 0.347) ^b^
Monounsaturated fatty acids (MUFA)		
Myristoleic acid (*cis*-9 C14:1)	0.001 (0.001; 0.001) ^a^	0.002 (0.002; 0.004) ^a^
Pentadecenoic acid (*cis*-10 C15:1)	0.002 (0.001; 0.003) ^a^	0.001 (0.001; 0.001) ^a^
Palmitoleic acid (*cis*-9 C16:1)	0.003 (0.002; 0.005) ^a^	0.013 (0.012; 0.014) ^a^
Heptadecenoic acid (*cis*-10 C17:1)	0.002 (0.002; 0.002) ^a^	0.004 (0.002; 0.013) ^a^
Elaidic acid (*trans*-9 C18:1)	0.001 (0.001; 0.005) ^a^	0.002 (0.002; 0.004) ^a^
Oleic acid (*cis*-9 C18:1)	0.015 (0.010; 0.019) ^a^	0.028 (0.013; 0.038) ^a^
Eicosanoic acid (*cis*-11 C20:1)	BDL	0.036 (0.025; 0.043)
Polyunsaturated fatty acids (PUFA)		
Linoleic acid (*trans*-9,12, *cis*-9,12 C18:2n-6)	0.048 (0.016; 0.069) ^a^	0.009 (0.003; 0.023) ^a^
γ-linolenic acid (*cis*-6,9,12 C18:3n-6)	0.006 (0.003; 0.028) ^a^	0.014 (0.013; 0.018) ^a^
α-linolenic acid (*cis*-9,12,15 C18:3n-3)	0.257 (0.101; 0.616) ^a^	0.169 (0.126; 0.175) ^a^

BDL, below detection level. Different characters (a, b) in lines indicate significant differences between HYP and CON (*p* < 0.05) according to the Mann–Whitney U test.

**Table 3:** Ratio of free fatty acids in blood serum of dairy cows under hyperthermia conditions (HYP) and in the control group (CON).

Indicator	HYP		CON		*p*-Value between Groups
	μg/μL	% of Total Fatty Acids	μg/μL	% of Total Fatty Acids	
Free fatty acids (FFA)	17.749	100.0	11.946	100.0	0.2447
Σ saturated fatty acids (SFA)	12.490	70.37	10.158	85.03	0.2291
Σ unsaturated fatty acids (UFA)	5.259	29.63	1.788	14.97	0.1105
Σ monounsaturated fatty acids (MUFA)	0.931	5.25	0.399	3.34	0.0059
Σ polyunsaturated fatty acids (PUFA)	4.328	24.38	1.389	11.63	0.1559
including, Σ n-3 PUFA	0.037	0.2	0.073	0.6	0.1381
Σ n-6 PUFA	2.894	16.3	0.146	1.2	0.2354
UFA/SFA ratio	0.42		0.18		0.0525
n-6/n-3 PUFA ratio	78.2		2.0		0.1663

## Data Availability

Data is available on request from the authors.
